# Identification of Superior Barley Genotypes Using Selection Index of Ideal Genotype (SIIG)

**DOI:** 10.3390/plants12091843

**Published:** 2023-04-29

**Authors:** Hassan Zali, Ali Barati, Alireza Pour-Aboughadareh, Ahmad Gholipour, Shirali Koohkan, Akbar Marzoghiyan, Jan Bocianowski, Henryk Bujak, Kamila Nowosad

**Affiliations:** 1 Crop and Horticultural Science Research Department, Fars Agricultural and Natural Resources Research and Education Center, Agricultural Research, Education and Extension Organization (AREEO), Darab P.O. Box 71558-63511, Iran; hzali90@yahoo.com; 2 Seed and Plant Improvement Institute, Agricultural Research, Education and Extension Organization (AREEO), Karaj P.O. Box 31587-77871, Iran; 3 Crop and Horticultural Science Research Department, Golestan Agricultural and Natural Resources Research and Education Center, Agricultural Research, Education and Extension Organization (AREEO), Gonbad P.O. Box 49156-77555, Iran; ahmadgholipour@yahoo.com; 4 Crop and Horticultural Science Research Department, Sistan Agricultural and Natural Resources Research and Education Center, Agricultural Research, Education and Extension Organization (AREEO), Zabol P.O. Box 98616-44534, Iran; koohkan182@gmail.com; 5 Crop and Horticultural Science Research Department, Khuzestan Agricultural and Natural Resources Research and Education Center, Agricultural Research, Education and Extension Organization (AREEO), Ahvaz P.O. Box 61335-3341, Iran; a.marzooghian@areeo.ac.ir; 6 Department of Mathematical and Statistical Methods, Poznań University of Life Sciences, Wojska Polskiego 28, 60-637 Poznań, Poland; jan.bocianowski@up.poznan.pl; 7 Department of Genetics, Plant Breeding and Seed Production, Wrocław University of Environmental and Life Sciences, Grunwaldzki 24A, 53-363 Wrocław, Poland; henryk.bujak@upwr.edu.pl; 8 Research Center for Cultivar Testing, Słupia Wielka 34, 63-022 Słupia Wielka, Poland

**Keywords:** heritability, multi-trait, REML, selection index, superior genotypes

## Abstract

The main objective of the study was to evaluate and select the superior barley genotypes based on grain yield and some pheno-morphological traits using a new proposed selection index (SIIG). For this purpose, one-hundred-eight pure and four local cultivars (Norouz, Auxin, Nobahar, and WB-97-11) were evaluated as reference genotypes in four warm regions of Iran, including Ahvaz, Darab, Zabol, and Gonbad, during the 2020–2021 cropping seasons. The results of REML analysis showed that the heritability of all traits (except plant height) was higher in Gonbad than in other environments, while the lowest values were estimated in Ahvaz and Zabol environments. In addition, among the measured traits, the thousand kernel weight and grain filling period showed the highest and lowest values of heritability (0.83 and 0.01, respectively). The results showed that the seed yield of genotypes 1, 108, 3, 86, 5, 87, 19, 16, 15, 56, and 18 was higher than the four reference genotypes, and, on the other hand, the SIIG index of these genotypes was greater than or equal to 0.60. Based on the SIIG discriminator index, 4, 8, 31, and 28 genotypes with values greater than or equal to 0.60 were identified as superior for Darab, Ahvaz, Zabol, and Gonbad environments, respectively. As a conclusion, our results revealed that the SIIG index has ideal potential to identify genotypes with high yield and desirable traits. Therefore, the use of this index can be beneficial in screening better genotypes in the early stages of any breeding program for any crop.

## 1. Introduction

Barley (*Hordeum vulgare* L.) has been recognized as one of the most compatible crops with production in many different regions of the world [1,2]. In addition, this cereal crop ranks fourth in the world in terms of economic importance after wheat, rice, and corn [3]. According to an FAO report, global barley production in 2019 was estimated at about 158.9 million tons, and the average production for Iran was estimated at 3.6 million tons [4].

Genetic diversity is critical to achieving the goals of breeding programs. Therefore, testing new genotypes in different breeding programs for favorable morphological traits can improve the final yield of released commercial genotypes [5,6]. Grain yield, as the most economic trait, has quantitative heritance and mainly depends on both genotypic and environmental factors. Therefore, a good alternative tool is indirect selection through other traits that have a high correlation with yield [7]. In recent years, several selection indexes based on multiple traits have been proposed, such as the selection index of ideal genotype (SIIG) [8], the multiple trait selection index (MTSI) [9], the genotype–ideotype distance index (MGIDI) [10], and the FAI-BLUP index [11], for selecting ideal genotypes with high yield and desirable growth traits.

Given the role of genetic diversity in the progress of breeding programs, the study of new barley genotypes with desirable morphological traits is undoubtedly one of the appropriate methods to improving yields, breeding, and introducing commercial cultivars, which ultimately leads to increased production in barley [12]. One method of indirect selection is selection based on appropriate selection indices [13]. In this way, a suitable index that is a combination of phenotypic values is determined and used to select the best genotype. However, this index should be correlated with the target trait (e.g., grain yield). In some cases, the purpose of selection is to select high-yielding genotypes with special traits, such as early maturity, dwarfism, etc. [13].

Selecting superior high–performing genotypes can be a difficult task for many traits simultaneously. Smith [14] and Hazel [15] first proposed a simultaneous selection index for plant breeding and animal breeding, respectively. The Smith–Hazel index is based on the selection of unknown genetic values. Thus, the use of genetic covariances and phenotypic values is necessary to determine how the weight vector should be selected to maximize the correlation of unknown phenotypic values and genetic values [16]. One of the difficulties in using the classic Smith–Hazel index is the lack of a method to weight traits of economic importance [17]. Genetic variation [18] and heritability have been assigned as relative economic weights, while, in other cases, they can be assigned randomly [19].

The SIIG index was first introduced by Zali et al. [8] to integrate various stability analysis methods. The SIIG method can be used to better rank and compare different genotypes and select the best genotypes, as well as determine the distances between genotypes and their clustering. Other features of the SIIG index can be used for other morphological traits, physiological traits, etc., which increases the efficiency of selection. Each genotype can be superior in terms of some index or trait; with increasing number of traits or indices, it becomes difficult to select the appropriate genotype. In the SIIG index, all indexes or traits become one index and it becomes easier to rank and identify superior genotypes [8,20]. The SIIG index allows selection of superior genotypes based on multiple traits, free from multicollinearity, and does not require assigning weights, as in the case of the SH index and its derived indexes.

Therefore, the purpose of the study was to evaluate and select the best barley genotypes on the basis of grain yield and a set of morphological traits using the SIIG index and to compare the effectiveness of this index with other proposed indexes in selecting superior genotypes.

## 2. Results

The summary of REML analysis for the number of days to heading (DHE), grain filling period (GFP), the number of days to physiological maturity (DMA), plant height (PLH), thousand kernels weight (TKW), and grain yield (YLD) is presented in Table 1. Based on the results, the heritability of all traits (except PLH) was higher in Gonbad than in other environments, and the lowest heritability of traits was observed in Ahvaz and Zabol. The heritability of DMA, DHE, TKW, GFP, and YLD traits in Gonbad was 0.89, 0.98, 0.70, 0.92, and 0.70, respectively. The highest heritability of PLH was observed in Darab (0.79). The lowest heritability of DHE and YLD traits was estimated in Ahvaz, while the lowest heritability of TKW and DMA traits was recorded for Zabol and Ahvaz. The Zabol environment showed the lowest heritability for PLH and GFP traits.

The results of REML analysis using the BLUP statistic for the studied genotypes are shown in Table 2. Accordingly, the highest heritability of genotypes was for TKW (0.83) and the lowest for GFP (0.01) and YLD (0.28). The genotype × environment interaction variance for DHE, DMA, GFP, and YLD traits was higher than the genotypic variance of these traits, while the opposite was true for PLH and TKW traits.

**Figure 1:**
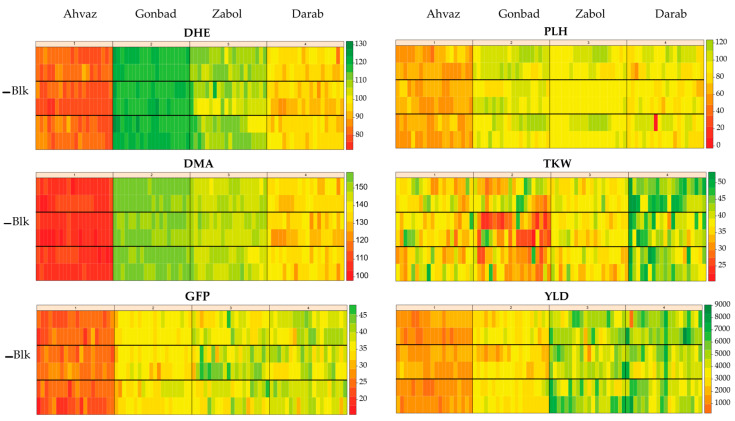
Heat maps of phenotypic variation of morpho-phonological traits of barley genotypes at different environments. DHE: number of days to heading; DMA: number of days to maturity; GFP: grain filling period; PLH: plant height; TKW: thousand kernel weight; YLD: grain yield.

Graphic phenotypic variation for the measured traits is shown Figure 1. The results indicated that the highest values of DMA, DHE, and PLH were recorded in the Moghan environment, while the lowest values were recorded in the Ahvaz environment. The lowest values of YLD and TKW were observed in Zabol and the highest values in the Darab environment. In addition, the Ahvaz and Darab environments showed the lowest and highest GFP, respectively.

Based on the results in Table 3, the highest grain yield was recorded for the Zabol (4805 kg ha^−1^) and Darab (4768 kg ha^−1^) environments, respectively. The Ahvaz and Zabol environments also showed the lowest and highest grain yield, respectively, and the difference between them was 3396 kg ha^−1^, which was higher than the average grain yield obtained in Moghan (3056 kg ha^−1^).

The SIIG selection index was used to select the best genotypes for each test environment and all warm environments in terms of grain yield and other measured traits (Table 4 and Table 5). According to the results of the SIIG index, genotypes 37, 107, 38, 71, 105, 104, 99, and 63 with the highest SIIG value (between 0.601 and 0.719) were identified as the best genotypes in the Ahvaz environment. The average grain yield and SIIG values of identified genotypes were higher than those of the reference genotypes (Table 3). In the Zabol environment, genotypes 54, 56, 1, 5, 18, 96, 4, 26, 108, and 16 were considered superior genotypes, along with the Nowruz cultivar with the highest SIIG index value (0.703–0.773). The range of the SIIG index for selected genotypes in the Darab environment was 0.659 and 0.611, and some genotypes, such as 86, 87, 1, and 108, were considered ideal genotypes compared to others. In addition, the Nowruz cultivar with a yield of 6957 kg ha^−1^ and a high SIIG value (0.611) was identified as the best reference genotype in this environment (Table 3). Genotypes 105, 80, 84, 36, 3, 99, and 107 with the highest SIIG index value (0.706–0.706) were the best genotypes in the Gonbad environment, respectively. The SIIG index values and grain yield of these genotypes were higher than those of all control genotypes (Table 3). Based on the average data from the four test environments, the identified superior genotypes were 86, 108, 3, 1, 87, 105, 99, 80, 4, 18, 5, 109, 97, 15, 82, 56, 16, and 23. The SIIG values for these genotypes ranged from 0.608 to 0.726 (Table 4).

**Figure 2:**
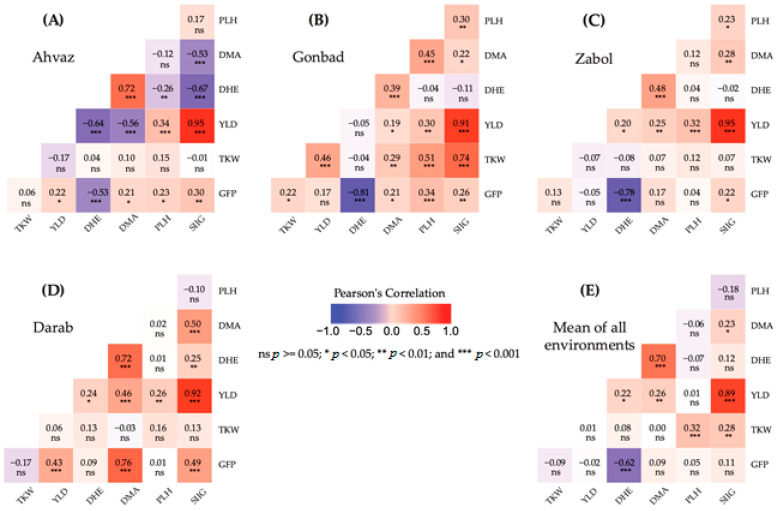
Correlation heat maps of morpho-phonological traits of barley genotypes at (**A**) Ahvaz, (**B**) Gonbad, (**C**) Zabol, and (**D**) Darab different environments and (**E**) mean of all environments. DHE: number of days to heading; DMA: number of days to maturity; GFP: grain filling period; PLH: plant height; TKW: thousand kernel weight; YLD: grain yield.

The results of the correlation of the SIIG index with the measured morpho-phenological traits are shown in Figure 2. Under Ahvaz conditions, the SIIG index showed a positive and significant correlation with YLD (0.95 **), while it negatively and significantly correlated with DMA (−0.67 **) and DHE (−0.53 **) (Figure 2A). In the Zabol environment, the SIIG index showed a significant positive relationship with YLD (0.95 **), DMA (0.28 **), PLH (0.23 *), and GFP (0.22 *) (Figure 2C). Based on the data obtained in the Darab environment, associations between SIIG index and YLD (0.92 **), DMA (0.50 **), and GFP (0.49 **) were positive and significant (Figure 2D).

In addition, a significant positive association was observed between the SIIG index with YLD (0.91 **) and TKW (0.74 **) in the Gonbad environment (Figure 2B). Based on the data, the selection index showed a positive and significant correlation with TKW and DMA traits (Figure 2E).

## 3. Discussion

The advantages of the REML method over classical methods are that it is highly efficient in augmentation designs and reduces the number of negative estimates of genetic parameters due to problems, especially inadequacy of experimental design, that arise in classical methods [21]. In the present study, our results showed that the heritability of most traits was higher in the Moghan environments compared to other test environments (Table 2). Despite the short flowering and maturation periods in the Darab environment compared to Gonbad and Zabol, the grain yield in the Darab environment was higher than in the other two environments (Figure 1), indicating ideal barley production conditions in Darab compared to the Gonbad and Zabol environments. This result can be appreciated by breeders and farmers to optimize barley yield in low-yielding environments in Iran’s tropical climate. Therefore, additional studies (such as stability tests) of selected genotypes can be used to develop high-yield genotypes or improve grain yield stability and productivity through appropriate breeding strategies.

As the results showed, groups 1 (genotypes with SIIG values greater than or equal to 0.7) and 2 (genotypes with SIIG values greater than or equal to 0.6 and less than 0.7) were superior genotypes based on the SIIG index (Table 5). Although all traits will eventually be reflected in grain yield, selection based on various traits can be effective in improving the process of breeding programs. One of the advantages of using the SIIG index is that all traits are considered and their effects are shared by genotype [8]. In other words, in this index, different traits will directly participate in the selection of genotypes [12]. Since irrigated crops are not usually exposed to water stress, dwarfism may be a key trait for lodging [22]. On the other hand, in breeding for drought stress tolerance, early maturity has an important role in improving grain yield [23,24,25]. Of course, it should be noted that early maturity will be useful when the grain filling period is not limited and the plant has the necessary opportunity to complete this period to prevent shrinkage and loss of grain weight [26]. Since the genotypes in the present study were tested in the warm environments of Iran, dwarfism, early maturity, and increasing the length of the grain-filling period were taken into account as selection criteria when choosing desirable genotypes. For other traits, such as TKW and YLD, the observation of high values was considered in the selection of desirable genotypes.

The different response of genotypes in the studied environments is due to the genotype × environment interaction, so, using the SIIG index, efforts were undertaken to introduce the best genotypes into additional experiments based on different traits in each environment. The results of correlating the SIIG index with various traits showed that, in all environments, there was a significant correlation between grain yield and SIIG index, indicating the effectiveness of the SIIG index in selecting high-yield genotypes. Traits with high variability will contribute more to the numerical value of the SIIG index [12]. Moreover, in each environment, the SIIG index introduced leading genotypes and showed their distance from other genotypes. The SIIG index is a selective model and is used to select the most ideal genotypes. In other words, using the SIIG index, different traits can be turned into a single index, and the selection of superior genotypes can be undertaken more reliably and accurately [20]. Another feature of the SIIG index is the integration of traits with different units [8].

The results of grouping genotypes based on SIIG index in Ahvaz showed that, as the SIIG value was lowered, the YLD and GFP values decreased and the DMA and DHE values increased, while no major changes were observed in TKW and PLH. Therefore, the use of the SIIG index led to the selection of genotypes with high yield and early maturity in Ahvaz. The results of the SIIG index in Darab showed that, with the reduction in SIIG, the amount of YLD and GFP increased, while the amount of DMA and DHE decreased. Therefore, in Darab, selection using the SIIG index led to the selection of high-yield but late-maturity genotypes. In Gonbad, as the SIIG value decreased, the amount of YLD, TKW, and GFP decreased, but not much change was observed in other traits. In Zabol, the results showed that, with decreasing SIIG value, YLD decreased, but no significant changes were observed in other traits. In general, the results of grouping genotypes in terms of the traits studied using the SIIG index in all environments showed that, as the SIIG value increased, YLD increased, but other traits increased or decreased due to correlation with YLD. Therefore, group 1 genotypes (0.70 ≤ SIIG) in any environment are superior genotypes that can outperform other genotypes in terms of YLD and other traits.

## 4. Materials and Methods

### 4.1. Genetic Materials and Setup Experiments

In this study, 108 pure barley genotypes were used along with 4 check varieties (cv. Nooruz, cv. Auxin, cv. Nobahar, and WB-97-11). This set of barley genotypes is derived from hybridization between local Iranian cultivars and international genetic materials obtained from ICARDA’s national barley breeding programs in SPII, Karaj, Iran. Nooruz, Auxin, and Nobahar are new and improved cultivars with high yield potential and excellent adaptability in different regions of warm Iran. Therefore, they were chosen as a reference for evaluating new genotypes. The experiment was performed at the following four locations: Gonbad (37°15′00″ N 55°10′02″ E), Zabol (31°01′43″ N 61°30′04″ E), Darab (28°45′07″ N 54°32′40″ E), and Ahvaz (31°19′13″ N 48°40′09″ E) during the 2020–2021 growing seasons. The meteorological characteristics of each environment are shown in Table 6.

The studied genotypes were planted in six lines along 6 m at a distance of 15 cm from each other on December 6. Seed consumption was determined by 300 seeds per square meter and thousand kernel weight for each genotype. Seeds were sown using an experimental plot planter (Wintersteiger, Ried, Austria). The fertilizer composition was 32 kg ha^−1^ nitrogen (twice), and di-ammonium phosphate and potassium sulfate were 100 and 50 kg ha^−1^, respectively (before planting). After the removal of perimeter plants, all experimental plots were harvested with an experimental grain harvester (Wintersteiger, Ried, Austria). The traits studied were the number of days to heading (DHE), days to maturity (DMA), plant height (PLH), thousand kernel weight (TKW), and grain yield (YLD).

### 4.2. Steps to Calculate the SIIG Index

#### 4.2.1. Formation of Data Matrix

Depending on the number of genotypes and the different traits measured, the data matrix was formed as the following equation (matrix D).

(1) $$D=\left[\begin{array}{ccc} \begin{array}{cc} \begin{array}{c} x_{11}\\ x_{21} \end{array} & \begin{array}{c} x_{12}\\ x_{22} \end{array} \end{array} & \cdots & \begin{array}{c} x_{1m}\\ x_{2m} \end{array}\\ \vdots & \ddots & \vdots \\ \begin{array}{cc} x_{n1} & x_{n2} \end{array} & \cdots & x_{\mathrm{nm}} \end{array}\right]$$

where *x*_ij_ is the value of the *i*th genotype (*i* = 1, 2,…, *n*) in relation to the *j*th trait (*j* = 1, 2, …, *m*).

#### 4.2.2. Converting the Primary Data Matrix (Matrix D) to a Normal Matrix (Matrix R)

The following relation is used to normalize the row data (without unifying the data):

(2) $$r_{ij}=\frac{x_{ij}}{\sqrt{\sum_{i=1}^{n}x_{ij}^{2}}}$$

(3) $$R=\left[\begin{array}{ccc} \begin{array}{cc} \begin{array}{c} r_{11}\\ r_{21} \end{array} & \begin{array}{c} r_{12}\\ r_{22} \end{array} \end{array} & \cdots & \begin{array}{c} r_{1m}\\ r_{2m} \end{array}\\ \vdots & \ddots & \vdots \\ \begin{array}{cc} r_{n1} & r_{n2} \end{array} & \cdots & r_{\mathrm{nm}} \end{array}\right]$$

#### 4.2.3. Finding the Ideal Genotype and Non-Ideal (Weak) Genotype for Each Trait

At this stage, according to the type of trait and the researcher’s opinion for each trait, the best (ideal) and the weakest (non-ideal) genotypes were selected. For example, in terms of grain yield, the maximum yield of a given genotype was considered the ideal value and the non-ideal value the lowest. In the case of days to maturity (DMA), minimum values are favorable.

#### 4.2.4. Calculating the Distance from Ideal Genotypes (d_i_^+^) and Non-Ideal Genotypes (d_i_^−^)

The distance coefficients from ideal genotypes (d_i_^+^) and weak genotypes (d_i_ were estimated based on the following relations.

(4) $$d_{i}^{+}=\sqrt{\sum_{j=1}^{m}{\left(r_{\mathrm{ij}}-r_{j}^{+}\right)}^{2}}\;i=1,\;\ldots \;,\;n$$

(5) $$d_{i}^{-}=\sqrt{\sum_{j=1}^{m}{\left(r_{\mathrm{ij}}-r_{j}^{-}\right)}^{2}}\;i=1,\;\ldots \;,\;n$$

where *r*_ij_ is the normalized value of *i*th genotype (*i* = 1, 2,…, *n*) in terms of *j*th trait (*j* = 1, 2, …, *m*). *r*_j_^+^ and *r*_j_^−^ indicated the normalized values of ideal genotypes and weak genotypes for each *j*th trait, respectively.

#### 4.2.5. Calculating the Ideal Genotype Selection Index (SIIG)

In the last step, Equation (6) shows the ideal genotype selection index for genotype:

(6) $${\mathrm{SIIG}}_{i}=\frac{d_{i}^{-}}{d_{i}^{+}+d_{i}^{-}}\;i=1,\;2\ldots ,\;n,\;0\leq {\mathrm{SIIG}}_{i}\leq 1$$

The SIIG_i_ value varies from 0 to 1, and genotypes with SIIG ≈ 1are selected as superior genotypes in terms of grain yield and other measured traits. All R scripts to calculate this index are shown in Appendix A.

### 4.3. Statistical Data Analysis

The experiment data were subjected to the calculation of analysis of variance based on the REML model using ACBD-R software [27]. Correlation analyses and heatmaps were performed using package ‘metan’ [28].

## 5. Conclusions

Overall, the SIIG index results in Darab, Ahvaz, Zabol, and Gonbad showed that genotypes 4, 8, 31, and 28 with SIIG values greater than or equal to 0.600 (0.60 ≤ SIIG) can be identified as superior genotypes for grain yield and other phonological traits. Since grain yield and related traits are inherited quantitatively, it is necessary to consider all yield-related traits to identify ideal genotypes in any breeding programs. Our results showed that the SIIG index has ideal potential to identify high-yielding genotypes with desirable traits. Therefore, the use of this index can be beneficial in screening the superior genotypes in the early steps of any breeding program for any crop.

## Figures and Tables

**Table 1:** Estimated genetic and residual variances and heritability values for measured morpho-phonological traits across different warm environments of Iran.

Environment	δ^2^g	δ^2^Res	He^2^	δ^2^g	δ^2^Res	He^2^	δ^2^g	δ^2^Res	He^2^
		DHE			PLH			DMA	
Ahvaz	0.313	0.205	0.605	0.468	0.198	0.703	0.303	0.188	0.617
Gonbad	0.778	0.012	0.985	0.314	0.114	0.733	0.638	0.078	0.892
Zabol	0.422	0.213	0.665	0.000	0.052	0.000	0.344	0.209	0.622
Darab	0.723	0.121	0.857	0.737	0.194	0.792	0.692	0.142	0.830
		**TKW**			**GFP**			**YLD**	
Ahvaz	0.000	0.016	0.000	0.219	0.167	0.568	0.203	0.209	0.492
Gonbad	0.412	0.173	0.705	0.699	0.059	0.922	0.373	0.160	0.700
Zabol	0.000	0.011	0.000	0.000	0.011	0.000	0.187	0.167	0.529
Darab	0.465	0.216	0.683	0.794	0.071	0.918	0.413	0.204	0.670

DHE: number of days to heading; DMA: number of days to maturity; GFP: grain filling period; PLH: plant height; TKW: thousand kernel weight; YLD: grain yield.

**Table 2:** Results of REML analysis for measured morpho-phonological traits in 108 genotypes of barley across 4 warm environments of Iran.

Statistic	DHE	DMA	GFP	PLH	TKW	YLD
Heritability	0.60	0.53	0.01	0.67	0.83	0.28
Genotype variance	1.97	0.54	0.011	20	13.3	5861
Gen × Loc variance	3.74	0.79	3.02	14	0	24,716
Avg Std Err (BLUP/BLUE)	2.03	1.02	1.00	5.67	2.90	67,320
Grand mean genotypes	101.8	135.7	32.5	84.6	38.4	3750
Grand mean check	102.5	136.1	32.3	84.6	40.8	3783
Avg Std Err difference genotype	0.85	0.76	0.88	3.29	2.37	133
LSD Genotypes	1.68	1.50	1.73	6.48	4.68	262
Avg Std Err difference check	0.61	0.79	0.68	2.14	1.48	161
LSD check	1.20	1.55	1.34	4.22	2.91	317
n Environments	4	4	3	3	2	4

DHE: number of days to heading; DMA: number of days to maturity; GFP: grain filling period; PLH: plant height; TKW: thousand kernel weight; YLD: grain yield.

**Table 3:** Grain yield, SIIG index values, and ranking pattern of genotypes based on SIIG index in different test environments.

Genotypes	Grain Yield (kg ha^−1^)				Ahvaz		Gonbad		Zabol		Darab	
	Ahvaz	Gonbad	Zabol	Darab	SIIG	Rank	SIIG	Rank	SIIG	Rank	SIIG	Rank
1	850	3183	7138	7780	0.303	103	0.503	59	0.758	3	0.612	3
2	1200	3475	5583	5313	0.341	93	0.540	49	0.535	53	0.490	32
3	1050	4400	6708	6020	0.372	78	0.731	5	0.682	14	0.528	14
4	1100	3722	5583	5220	0.366	81	0.696	8	0.709	7	0.460	50
5	1500	3538	6694	5497	0.463	43	0.566	41	0.735	4	0.498	29
6	1150	2772	5416	3787	0.363	83	0.536	51	0.614	29	0.395	86
7	1300	3605	5291	3780	0.363	82	0.639	16	0.549	49	0.351	102
8	1150	2982	4944	4370	0.440	50	0.472	72	0.498	70	0.434	64
9	1400	2903	3930	6200	0.447	47	0.457	77	0.394	97	0.530	12
10	800	2860	3416	3053	0.266	108	0.465	74	0.372	103	0.337	106
11	1100	3408	5805	3710	0.396	64	0.571	37	0.646	19	0.402	81
12	1100	2977	4416	4283	0.328	98	0.497	63	0.474	74	0.454	52
13	1800	2752	4000	4947	0.543	19	0.420	83	0.375	102	0.450	54
14	1550	2667	4569	5170	0.460	44	0.463	75	0.458	80	0.450	55
15	1900	3135	6513	4930	0.577	11	0.500	62	0.683	13	0.465	46
16	1600	3057	6527	5390	0.486	36	0.481	68	0.703	10	0.505	25
17	1700	2787	5180	3320	0.503	29	0.544	47	0.534	55	0.331	108
18	1650	3048	7263	4367	0.539	20	0.505	56	0.722	5	0.433	65
19	1300	3418	5652	6317	0.391	67	0.489	67	0.578	39	0.563	7
20	1600	2478	4305	6300	0.473	40	0.370	98	0.413	92	0.510	21
21	1300	2782	3458	5920	0.387	69	0.467	73	0.406	93	0.509	23
22	700	3160	3555	5820	0.249	111	0.512	55	0.362	104	0.489	34
23	1000	3058	6138	5457	0.311	101	0.553	45	0.679	15	0.491	31
24	1100	3363	4500	4360	0.337	94	0.578	33	0.495	71	0.416	71
25	800	3403	5027	3683	0.250	110	0.576	35	0.558	45	0.578	6
26	600	3647	6347	4240	0.267	107	0.652	13	0.707	8	0.412	73
27	950	3638	4569	5840	0.367	80	0.618	22	0.488	72	0.501	28
28	1500	2710	4291	4420	0.413	61	0.419	84	0.435	86	0.394	87
29	1200	3667	6305	4600	0.336	95	0.577	34	0.628	24	0.425	67
30	1200	2932	4486	4000	0.355	87	0.472	71	0.467	78	0.399	85
31	1400	2882	5222	3833	0.360	86	0.428	79	0.529	58	0.362	95
32	1100	2665	3555	3617	0.296	104	0.552	46	0.422	88	0.353	101
33	1700	3255	3944	4747	0.517	25	0.493	65	0.468	77	0.423	68
34	1400	3712	2680	3737	0.433	52	0.529	52	0.330	106	0.384	90
35	1450	3905	2805	4270	0.442	49	0.591	30	0.337	105	0.407	76
36	1500	4687	4333	3170	0.474	39	0.740	4	0.520	62	0.357	98
37	2700	2997	3611	4140	0.719	1	0.425	81	0.422	89	0.405	79
38	2000	3482	4472	3063	0.648	3	0.676	9	0.574	40	0.342	105
39	1800	3067	4208	3107	0.555	18	0.568	40	0.523	61	0.306	110
40	1800	3367	4750	3540	0.583	10	0.635	20	0.608	30	0.355	99
41	1800	3720	4916	4010	0.560	15	0.617	23	0.594	32	0.385	89
42	1700	3140	3805	3227	0.537	21	0.504	57	0.446	81	0.347	104
43	1250	3502	4472	3080	0.423	55	0.543	48	0.532	57	0.326	109
44	1000	3183	5152	4667	0.386	70	0.560	43	0.563	43	0.480	39
45	1750	3302	4222	4403	0.557	17	0.492	66	0.516	67	0.460	51
46	1400	2688	5277	4420	0.431	53	0.351	99	0.616	27	0.406	78
47	1200	2622	4388	3150	0.395	65	0.389	90	0.502	69	0.333	107
48	1300	3585	4875	3993	0.416	59	0.538	50	0.580	38	0.400	83
49	1300	1888	4027	3567	0.446	48	0.232	111	0.460	79	0.351	103
50	1700	2158	3166	4330	0.483	37	0.306	106	0.380	100	0.410	75
51	1600	2713	4819	5320	0.483	38	0.503	58	0.592	33	0.486	36
52	1400	2688	5472	3983	0.416	58	0.378	94	0.667	18	0.359	97
53	1400	2663	5000	3947	0.418	56	0.399	89	0.581	37	0.378	91
54	1600	2267	7319	4137	0.514	26	0.315	103	0.773	1	0.413	72
55	1800	2307	5638	5610	0.501	31	0.382	92	0.616	28	0.540	11
56	1800	2202	6333	5997	0.534	22	0.307	105	0.761	2	0.527	15
57	1550	1660	5666	4860	0.492	32	0.298	108	0.633	23	0.436	60
58	1250	2120	5236	6410	0.374	77	0.333	101	0.564	42	0.560	8
59	1100	2142	5069	4317	0.376	75	0.370	97	0.618	25	0.399	84
60	1100	1163	3888	3337	0.346	90	0.263	109	0.377	101	0.353	100
61	1500	2083	4541	3933	0.451	45	0.332	102	0.518	64	0.412	74
62	1400	2660	5805	5430	0.393	66	0.426	80	0.674	17	0.510	22
63	2100	2090	3138	6087	0.601	8	0.315	104	0.326	107	0.518	18
64	1800	3283	3319	5323	0.567	12	0.648	15	0.417	91	0.490	33
65	1300	3142	5333	3757	0.412	62	0.528	53	0.585	35	0.377	93
66	1700	3480	3736	3970	0.532	23	0.586	31	0.420	90	0.393	88
67	1800	3452	4263	4653	0.566	13	0.570	39	0.471	75	0.454	53
68	1650	2953	4527	4600	0.491	33	0.455	78	0.516	66	0.418	70
69	1800	2937	3750	5110	0.509	28	0.408	88	0.399	94	0.466	45
70	1700	2745	5583	4260	0.490	34	0.415	85	0.635	21	0.443	57
71	2200	2048	4805	3383	0.638	4	0.256	110	0.566	41	0.377	92
72	1900	2312	4263	6017	0.591	9	0.385	91	0.446	82	0.595	5
73	1200	2690	4902	5223	0.361	84	0.495	64	0.562	44	0.552	9
74	950	3397	5180	4000	0.308	102	0.638	18	0.589	34	0.485	38
75	1450	4147	4680	4017	0.439	51	0.619	21	0.545	51	0.403	80
76	1000	3783	4763	4630	0.336	97	0.649	14	0.532	56	0.443	58
77	850	3513	5250	4550	0.275	106	0.637	19	0.557	46	0.407	77
78	850	3347	4486	5797	0.281	105	0.614	25	0.476	73	0.505	26
79	1200	3347	3583	4807	0.377	74	0.607	28	0.395	96	0.471	42
80	1000	4172	4638	5370	0.353	89	0.752	2	0.513	68	0.501	27
81	1400	3302	1944	5190	0.414	60	0.615	24	0.258	111	0.466	44
82	1200	2827	6152	4943	0.368	79	0.565	42	0.699	11	0.479	40
83	750	3143	4111	4950	0.257	109	0.638	17	0.441	83	0.462	49
84	1000	3740	3097	4847	0.342	92	0.744	3	0.399	95	0.435	61
85	900	3553	3888	5440	0.411	63	0.654	12	0.440	84	0.527	16
86	1300	3955	4250	8360	0.449	46	0.656	11	0.470	76	0.659	1
87	1500	3702	4902	7020	0.489	35	0.608	27	0.537	52	0.621	2
88	1350	2207	4944	5600	0.378	73	0.348	100	0.555	47	0.487	35
89	1200	2683	5833	4927	0.376	76	0.408	87	0.697	12	0.467	43
90	1300	2762	5986	4707	0.430	54	0.459	76	0.644	20	0.462	48
91	1300	2892	4152	450	0.418	57	0.480	70	0.438	85	0.198	111
92	1200	2583	4347	4660	0.360	85	0.423	82	0.424	87	0.435	63
93	1000	2147	3125	5700	0.355	88	0.302	107	0.297	110	0.495	30
94	800	2513	3180	6187	0.312	100	0.381	93	0.304	109	0.518	19
95	950	2257	5208	6017	0.343	91	0.372	96	0.535	54	0.509	24
96	1050	2442	6444	5260	0.326	99	0.374	95	0.712	6	0.463	47
97	1000	3682	6208	5000	0.336	96	0.609	26	0.678	16	0.474	41
98	1200	2650	5833	4803	0.381	71	0.415	86	0.603	31	0.439	59
99	1900	3548	4180	5883	0.601	7	0.713	6	0.519	63	0.513	20
100	1750	2833	4916	5160	0.501	30	0.501	61	0.516	65	0.430	66
101	1350	2807	5166	4877	0.388	68	0.501	60	0.550	48	0.448	56
102	1850	3192	5000	4267	0.509	27	0.599	29	0.527	59	0.372	94
103	2000	3752	5083	3820	0.518	24	0.662	10	0.526	60	0.359	96
104	2100	2735	6222	4933	0.613	6	0.481	69	0.617	26	0.435	62
105	2400	4242	5305	6443	0.637	5	0.761	1	0.547	50	0.526	17
106	2100	3108	3805	4220	0.560	16	0.582	32	0.391	98	0.401	82
107	2000	4103	3250	3800	0.653	2	0.706	7	0.383	99	0.422	69
108	1950	3206	6425	6957	0.562	14	0.559	44	0.706	9	0.611	4
109	1500	3450	4967	6107	0.470	42	0.570	38	0.581	36	0.529	13
110	1200	3153	5814	4671	0.380	72	0.576	36	0.635	22	0.485	37
111	1617	3217	3194	7107	0.471	41	0.519	54	0.310	108	0.544	10

**Table 4:** Mean and rank of SIIG index for different traits in the investigated barley genotypes across different warm environments of Iran.

Genotypes	DHE	Rank	DMA	Rank	GFP	Rank	PLH	Rank	TKW	Rank	YLD	Rank	SIIG	Rank
86	101	45	134	28	33	95	75.0	3	38.0	48	4466	5	0.726	1
108	106	108	137	91	31	110	85.1	36	40.8	23	4634	2	0.705	2
3	103	75	137	83	34	75	85.8	44	38.5	44	4545	4	0.700	3
1	106	108	139	108	33	95	85.8	44	37.0	45	4738	1	0.687	4
87	101	45	134	28	33	95	74.3	2	35.6	78	4281	7	0.677	5
105	102	58	135	51	33	95	95.3	98	39.0	35	4598	3	0.662	6
99	97	5	135	49	38	4	83.8	28	38.9	37	3878	20	0.656	7
80	103	75	137	88	35	52	87.0	61	43.1	9	3795	25	0.635	8
4	100	31	135	44	36	25	96.8	103	46.3	2	3906	18	0.634	9
18	101	45	135	45	34	75	81.0	12	36.1	75	4082	12	0.634	10
5	104	97	137	84	34	75	82.0	19	34.4	92	4307	6	0.632	11
109	100	31	136	69	36	25	89.4	79	37.9	49	4006	13	0.626	12
97	101	45	135	49	35	52	89.3	78	38.7	39	3972	15	0.623	13
15	101	45	135	44	34	75	82.3	20	34.7	87	4120	10	0.623	14
82	102	58	138	106	36	25	92.5	91	43.7	8	3781	27	0.616	15
56	99	22	138	103	39	1	89.8	81	35.3	81	4083	11	0.614	16
16	102	58	135	44	33	95	82.5	22	35.3	82	4143	9	0.613	17
23	103	75	137	85	35	52	95.5	99	42.7	10	3913	17	0.608	18
110	107	110	139	111	33	95	81.3	13	41.4	20	3710	30	0.599	19
85	99	22	136	66	37	11	76.3	4	39.7	28	3445	58	0.598	20
19	104	97	136	61	33	95	84.3	29	34.1	96	4172	8	0.598	21
27	103	75	138	100	35	52	86.5	52	40.0	25	3749	29	0.594	22
55	100	31	134	23	35	52	86.5	52	37.3	64	3839	21	0.584	23
72	102	58	136	63	34	75	79.8	7	39.4	30	3623	37	0.583	24
62	100	31	136	62	37	11	84.5	30	34.7	88	3824	23	0.580	25
54	99	22	132	10	33	95	85.3	37	36.9	70	3831	22	0.578	26
58	103	75	137	86	34	75	85.0	35	37.7	53	3754	28	0.575	27
64	98	11	132	10	35	52	89.0	76	44.6	4	3431	62	0.573	28
73	102	58	135	48	33	95	81.8	15	42.0	16	3504	53	0.572	29
51	98	11	135	48	38	4	86.3	49	37.3	63	3613	40	0.566	30
26	104	97	137	86	34	75	87.3	64	39.2	31	3708	31	0.565	31
96	102	58	134	29	33	95	92.0	87	39.5	29	3799	24	0.565	32
11	105	104	139	109	34	75	79.3	6	38.7	38	3506	52	0.557	33
104	101	45	135	50	34	75	97.0	104	36.9	69	3998	14	0.554	34
40	95	1	132	9	37	11	91.5	84	43.9	6	3364	72	0.554	35
74	103	75	135	48	32	105	80.5	10	42.6	11	3382	69	0.551	36
41	99	22	134	23	36	25	92.8	92	39.9	27	3612	41	0.549	37
44	101	45	138	102	37	11	83.0	24	37.5	57	3501	54	0.545	38
90	102	58	137	89	35	52	87.8	70	37.3	62	3689	32	0.545	39
38	96	2	132	8	35	52	89.8	81	46.4	1	3254	82	0.543	40
36	96	2	134	22	38	4	87.5	67	38.5	42	3422	63	0.541	41
25	101	45	137	85	36	25	71.8	1	38.3	46	3228	88	0.540	42
107	97	5	130	1	34	75	83.3	26	42.0	15	3288	80	0.538	43
89	100	31	137	88	37	11	86.5	52	35.3	80	3661	36	0.538	44
78	104	97	136	65	32	105	86.5	52	39.1	33	3620	39	0.530	45
95	102	58	134	29	32	105	92.3	88	40.7	24	3608	43	0.528	46
76	99	22	134	27	35	52	86.8	58	36.6	72	3544	48	0.524	47
67	100	31	137	87	36	25	87.5	67	37.3	65	3542	49	0.524	48
9	103	75	134	22	31	110	86.5	52	38.6	41	3608	42	0.522	49
29	103	75	138	101	35	52	95.8	100	34.3	95	3943	16	0.521	50
70	102	58	136	63	34	75	87.3	64	37.5	58	3572	46	0.517	51
2	105	104	137	82	32	105	84.5	30	32.3	108	3893	19	0.517	52
6	102	58	135	44	33	95	81.8	15	41.0	21	3281	81	0.516	53
84	104	97	137	88	34	75	84.5	30	43.7	7	3171	92	0.514	54
75	99	22	134	26	35	52	85.5	40	34.9	85	3573	45	0.513	55
48	98	11	133	15	35	52	80.8	11	34.5	90	3438	59	0.511	56
111	103	75	136	69	34	75	94.7	97	35.9	76	3784	26	0.510	57
45	98	11	135	47	37	11	79.8	7	33.5	102	3419	64	0.505	58
8	102	58	134	21	32	105	81.8	15	38.6	40	3361	74	0.504	59
77	103	75	136	64	33	95	93.5	95	40.0	26	3541	50	0.499	60
79	102	58	137	88	35	52	90.5	83	42.5	12	3234	86	0.498	61
98	103	75	138	107	35	52	92.3	88	35.7	77	3622	38	0.498	62
21	100	31	134	22	35	52	86.8	58	37.6	54	3365	71	0.496	63
14	103	75	134	22	32	105	88.3	73	38.4	45	3489	56	0.489	64
100	103	75	136	68	34	75	100.8	108	39.2	32	3665	34	0.488	65
65	102	58	136	62	34	75	84.5	30	37.5	56	3383	68	0.487	66
103	100	31	134	29	34	75	108.5	111	42.0	14	3664	35	0.487	67
101	102	58	137	90	35	52	97.8	105	39.1	34	3550	47	0.487	68
83	103	75	136	66	33	95	88.5	74	42.3	13	3239	85	0.483	69
88	100	31	135	48	35	52	87.5	67	34.0	97	3525	51	0.481	70
24	105	104	139	110	34	75	94.0	96	41.8	18	3331	77	0.481	71
57	101	45	136	62	35	52	83.5	27	34.3	93	3434	60	0.481	72
102	100	31	134	29	34	75	107.8	110	42.0	17	3577	44	0.476	73
20	104	97	137	84	32	105	85.5	40	31.8	109	3671	33	0.474	74
12	103	75	138	99	35	52	82.3	20	37.0	66	3194	91	0.469	75
7	102	58	132	5	30	111	91.5	84	39.0	36	3494	55	0.469	76
68	98	11	134	24	36	25	86.8	58	33.3	103	3433	61	0.469	77
33	98	11	135	46	37	11	92.3	88	34.9	84	3411	65	0.467	78
59	100	31	134	23	35	52	82.5	22	37.5	55	3157	94	0.462	79
46	100	31	132	10	33	95	86.0	47	33.6	101	3446	57	0.461	80
39	97	5	131	2	34	75	100.3	107	45.5	3	3045	99	0.458	81
106	103	75	136	68	33	95	95.8	100	40.9	22	3308	79	0.452	82
52	97	5	133	16	36	25	93.0	94	34.5	89	3386	67	0.451	83
66	98	11	131	2	33	95	85.8	44	37.4	59	3222	89	0.449	84
53	100	31	133	16	34	75	86.3	49	35.6	79	3253	83	0.448	85
63	102	58	136	62	35	52	87.3	64	33.7	100	3354	75	0.448	86
17	101	45	131	2	31	110	85.5	40	37.9	50	3247	84	0.446	87
37	98	11	132	7	34	75	85.5	40	33.1	104	3362	73	0.445	88
22	103	75	135	46	33	95	89.0	76	36.6	71	3309	78	0.442	89
13	103	75	133	15	31	110	86.0	47	35.0	83	3375	70	0.439	90
69	101	45	136	62	35	52	86.5	52	31.3	110	3399	66	0.433	91
43	97	5	133	15	36	25	88.5	74	37.3	60	3076	98	0.428	92
61	98	11	134	23	35	52	78.8	5	32.8	106	3014	100	0.423	93
94	103	75	134	29	32	105	88.0	71	37.9	51	3170	93	0.423	94
92	105	104	136	67	32	105	88.0	71	37.3	61	3198	90	0.414	95
42	96	2	132	10	36	25	83.0	24	34.3	94	2968	102	0.414	96
81	103	75	138	105	35	52	92.8	92	41.5	19	2959	103	0.412	97
71	99	22	134	25	35	52	84.5	30	32.9	105	3109	96	0.407	98
30	100	31	136	61	36	25	91.8	86	34.7	86	3154	95	0.402	99
35	98	11	133	15	36	25	89.5	80	34.4	91	3108	97	0.399	100
32	97	5	132	6	35	52	101.0	109	43.9	5	2734	107	0.390	101
47	99	22	132	10	34	75	79.8	7	34.0	98	2840	105	0.390	102
28	101	45	134	22	33	95	97.8	105	37.0	68	3230	87	0.382	103
93	103	75	136	67	33	95	87.0	61	36.2	74	2993	101	0.378	104
31	102	58	136	61	34	75	96.5	102	32.7	107	3334	76	0.377	105
34	99	22	134	22	36	25	87.0	61	33.9	99	2882	104	0.371	106
49	101	45	136	61	35	52	86.3	49	36.5	73	2696	108	0.361	107
50	98	11	134	23	36	25	85.3	37	31.0	111	2839	106	0.357	108
10	104	97	137	84	33	95	85.3	37	38.3	47	2532	109	0.335	109
60	107	110	138	104	31	110	81.5	14	38.5	43	2372	110	0.323	110
91	103	75	135	49	32	105	81.8	15	37.8	52	2198	111	0.317	111

DHE: number of days to heading; DMA: number of days to maturity; GFP: grain filling period; PLH: plant height; TKW: thousand kernel weight; YLD: grain yield.

**Table 5:** Grouping of barley genotypes based on SIIG index and measured morpho-phonological traits across different environments.

SIIG Index	Environment	Groups	Number of Genotypes	Average of Groups					
				DHE (Day)	DMA (Day)	DMA (Day)	PLH (cm)	TKW (g)	YLD (kg h^−1^)
0.70 ≤ SIIG > 1.00		1	1	77	103	26	65	32	2700
0.60 ≤ SIIG > 0.70		2	7	78	104	25	66	37	2100
0.50 ≤ SIIG > 0.60	Ahvaz	3	23	79	104	25	68	37	1802
0.40 ≤ SIIG > 0.50		4	32	81	106	25	61	36	1436
0.00 ≤ SIIG > 0.40		5	48	84	108	24	64	37	1076
0.60 ≤ SIIG > 0.70		1	4	97	137	40	84	44	7529
0.50 ≤ SIIG > 0.60	Darab	2	23	96	136	40	90	44	5860
0.40 ≤ SIIG > 0.50		3	55	95	134	39	90	43	4722
0.00 ≤ SIIG > 0.40		4	28	94	130	36	90	43	3525
0.70 ≤ SIIG > 1.00		1	7	118	154	37	100	40	4127
0.60 ≤ SIIG > 0.70		2	21	118	154	36	103	39	3575
0.50 ≤ SIIG > 0.60	Gonbad	3	34	119	155	35	103	36	3216
0.40 ≤ SIIG > 0.50		4	26	119	154	35	100	31	2876
0.00 ≤ SIIG > 0.40		5	23	119	153	34	94	27	2224
0.70 ≤ SIIG > 1.00		1	10	109	148	39	97	38	6607
0.60 ≤ SIIG > 0.70		2	21	109	148	39	98	37	5819
0.50 ≤ SIIG > 0.60	Zabol	3	38	107	146	39	97	37	4904
0.40 ≤ SIIG > 0.50		4	24	108	146	38	95	37	4137
0.00 ≤ SIIG > 0.40		5	18	110	146	36	92	37	3306
0.70 ≤ SIIG > 1.00		1	3	103	136	33	82	39	4548
0.60 ≤ SIIG > 0.70		2	15	101	136	35	87	39	4107
0.50 ≤ SIIG > 0.60	Means	3	41	101	135	34	86	39	3592
0.40 ≤ SIIG > 0.50		4	40	101	135	34	90	37	3333
0.00 ≤ SIIG > 0.40		5	12	101	135	34	88	36	2813

DHE: number of days to heading; DMA: number of days to maturity; GFP: grain filling period; PLH: plant height; TKW: thousand kernel weight; YLD: grain yield.

**Table 6:** Monthly meteorological data in 2020–2021 cropping seasons in the warm environments of Iran.

Month	Darab				Ahvaz			
	Temperature (°C)			Rainfall (mm)	Temperature (°C)			Rainfall (mm)
	Min (°C)	Max (°C)	Mean (°C)		Min (°C)	Max (°C)	Mean (°C)	
October	16	33.4	24.7	0	20.4	39.5	30	0
November	16.2	27.3	18.7	7	14.8	31.3	23	21.5
December	7.6	20.9	14.2	40.6	13.1	21.4	17.2	30.9
January	2.2	19.4	10.8	2	7.2	21.2	14.2	0.9
February	4.5	22.8	13.7	4.1	9.7	23.7	16.7	17.6
March	9.9	24.6	17.2	17.2	12.4	25.4	18.9	5.6
April	13.6	31.5	22.6	0.9	17.9	33.8	25.8	0
May	18.4	34.8	22.6	1.9	24.5	41.8	32.9	0
June	23.3	42	32.7	0	28.8	46.2	37.5	0
Sum				73.7				76.5
	**Gonbad**				**Zabol**			
	**Temperature (°C)**			**Rainfall (mm)**	**Temperature (°C)**			**Rainfall (mm)**
	**Min (°C)**	**Max (°C)**	**Mean (°C)**		**Min (°C)**	**Max (°C)**	**Mean (°C)**	
October	8.1	32.6	20.4	30.4	15.4	29	18	0
November	1.5	37.2	19.4	16.5	8.7	24.1	16.4	2.6
December	−4	20	8	31.9	3.6	17.1	10.4	1.4
January	−3.1	29.6	13.3	31.8	−0.6	18.8	7.6	0
February	−3.9	30.1	13.1	24.5	3.7	22.7	13.2	0
March	−5.7	35.9	15.1	62.2	10	26.9	18.4	0
April	2.6	34.3	18.5	16.6	16	32.9	24.5	17.2
May	12.2	43.8	28	20	21.1	36.5	28.8	0
June	14.8	46.5	30.7	12.2	27.2	43.3	35.3	0
Sum				246.1				21.2

## Data Availability

The data in this manuscript are available from the corresponding author upon reasonable request.

## Appendix A

R program to calculate the selection index of ideal genotype (SIIG):

This program has been prepared for one-hundred-twelve genotypes and thirteen traits, where the high value of the first ten traits and the low value of the last three traits are ideal.

#read file

m.a <-read.table(“E:/Siig-index.txt”, header=TRUE, sep=““)

#####square array####

m.a2<- m.a^2

#sum of each columns####

m.b<-colSums (m.a2, na.rm = FALSE, dims = 1)

dim(m.b)<-c(1,13)

m.b<-m.b^0.5

m.b<-m.b[rep(1:nrow(m.b), times = 112), ]

#matrix c devide matrices

m.c<-m.a/m.b

#######make maximum.minimum1 matrix#############

max1 <-m.c[,1:10]

max1 <-apply(max1,2,max)

dim(max1)<-c(1,10)

max1<-max1[rep(1:nrow(max1), times = 112), ]

#******

min1 <-m.c[,11:13]

min1 <-apply(min1,2,min)

dim(min1)<-c(1,3)

min1<-min1[rep(1:nrow(min1), times = 112), ]

#***

maxmin1<-cbind(max1,min1)

####make maximum.minimum1 matrix#####

max2 <-m.c[,11:13]

max2 <-apply(max2,2,max)

dim(max2)<-c(1,3)

max2<-max2[rep(1:nrow(max2), times =112), ]

#******

min2 <-m.c[,1:10]

min2 <-apply(min2,2,min)

dim(min2)<-c(1,10)

min2<-min2[rep(1:nrow(min2), times = 112), ]

#***

maxmin2<-cbind(min2,max2)

#square maxmin matrix

tmaxmin1<-(m.c-maxmin1)^2

tmaxmin2<-(m.c-maxmin2)^2

#d+ matrix

dplus <-rowSums (tmaxmin1, na.rm = FALSE, dims = 1)

dplus<-dplus^0.5

dim(dplus)<-c(112,1)

#d- matrix

dminus <-rowSums (tmaxmin2, na.rm = FALSE, dims = 1)

dminus<-dminus^0.5

dim(dminus)<-c(112,1)

#####SIIG######

SIIG<-dminus/(dminus+dplus)

###write file###

print(dplus)

print(dminus)

print(SIIG)
